# Report from the first Tanzania Liver Cancer Conference: a call for action to unite in the fight against liver cancer in Sub-Saharan Africa, 17–18 March 2023, Dar es Salaam, Tanzania

**DOI:** 10.3332/ecancer.2023.1543

**Published:** 2023-05-02

**Authors:** Ally Mwanga, Eric Mbuguje, Jared Alswang, Nathan Brand, Andrew Swallow, Deogratius Beda Mwanakulya, Charles Pallangyo, Seif Wibonela, Eva Uiso, William Kitua, Saleh Pazi

**Affiliations:** † Dr Mwanga and Dr Mbuguje contributed equally and are designated co-first authors on this manuscript.; 1 Department of Surgery, Muhimbili University of Health and Allied Sciences, Dar es Salaam, Tanzania; 2 Department of Radiology and Imaging, Muhimbili National Hospital, Dar es Salaam, Tanzania; 3 Harvard Medical School, Boston, MA 02115, USA; 4 Department of Surgery, University of California, San Francisco, CA 94143, USA; 5 Department of Surgery, Muhimbili National Hospital, Dar es Salaam, Tanzania; 6 Department of Internal Medicine, Adult Oncology Unit, Muhimbili National Hospital, Dar es Salaam, Tanzania; 7 Clinical Research, Training and Consultancy Unit, Muhimbili National Hospital, Dar es Salaam, Tanzania; 8 Department of Internal Medicine, Medical Gastroenterology Unit, Muhimbili National Hospital, Dar es Salaam, Tanzania

**Keywords:** liver cancer, Tanzania, global cancer, surgical oncology, hepatocellular carcinoma

## Abstract

The first Tanzania Liver Cancer Conference (TLCC2023) took place on 17–18 March 2023 in Dar es Salaam, Tanzania with the aim of raising awareness among healthcare providers on the problem that liver cancer poses to the Tanzanian population and the urgent need to address this important issue. The conference focused on the following agenda items: 1) to build awareness among local healthcare providers on the status of liver cancer in Tanzania and the available diagnostic and management options, 2) to update Tanzanian healthcare providers on the current standard of care for liver cancer provided in developed countries and recent advancements in liver cancer care and 3) to promote an inclusive and multidisciplinary approach in research and the clinical care of patients with liver cancer in Tanzania. TLCC2023 was preceded by community-facing pre-conference activities, including screening 684 community members for hepatitis B virus free of charge. The conference was attended by 161 healthcare professionals from varying disciplines across Tanzania and abroad. TLCC2023 featured over 30 speakers from Tanzania, Kenya, Egypt, India and the United States that comprehensively covered a wide range of topics related to research and clinical care of liver cancer patients. A holistic and unified approach integrating both private and public sectors is vital in improving care for patients with liver cancer, and this was a common theme ingrained in the majority of presentations. Overall, the conference was well-received by attendees and knowledge assessment scores improved from 50% pre-conference to 75% post-conference (p < 0.001), demonstrating its educational value. As Tanzania’s first conference on the subject, TLCC2023 marked an important milestone in a united fight against liver cancer in the country and beyond.

## Introduction

On 17–18 March 2023 the Tanzania Liver Cancer Group (TLCG) hosted the first Tanzania Liver Cancer Conference (TLCC) in Dar es Salaam, Tanzania. TLCG is a multi-institutional team of surgeons, interventional radiologists, diagnostic radiologists, pathologists, gastroenterologists and medical oncologists that formed a working group in 2022 to address liver cancer in Tanzania through a multidisciplinary approach. The goal of the conference was to offer a comprehensive educational experience to Tanzanian healthcare providers involved in the care of patients with liver cancer, which is a high-burden, high-mortality disease in the region. Additionally, the conference was designed to raise local and international awareness of the scope of the problem and serve as a catalyst to unite an international, interdisciplinary community to address the problems through collaborative research and the advancement of care.

In total, 161 people attended the conference from various disciplines across Tanzania and abroad. The conference featured over 30 speakers from Tanzania, Kenya, Egypt, India and the United States (US). It provided a comprehensive overview of the state of liver cancer in Tanzania, updates on liver cancer diagnosis and treatment in high-income countries, and the promotion of collaborative research and the utilisation of treatment guidelines to advance liver cancer care in sub-Saharan Africa (SSA).

## Background on liver cancer in Tanzania

Liver cancer is the seventh most common cancer and the second most common cause of cancer-related mortality worldwide, totalling over 830,000 deaths each year [1]. The global burden of hepatocellular carcinoma (HCC) falls disproportionately on resource-limited settings, with 80% of cases occurring in low- and middle-income countries, particularly in SSA and East/Southeast Asia [2]. This disparity is especially troublesome in SSA given the dismal prognosis of HCC in the region with median survival times reported as short as 8 weeks from presentation [3]. Further, HCC in SSA commonly presents at a younger age, occurring in adults as young as 30 years old, robbing many of their prime and putting substantial strain on resource-limited health systems [3].

In Tanzania and elsewhere in East Africa, there is a paucity of population-based data available on the incidence of liver cancer. However, a nationwide study on cancer mortality in 39 hospitals throughout the country found that liver cancer is the third most common cause of cancer death, next to cancer of the cervix and cancer of the oesophagus [4]. It is estimated that HCC is responsible for 75%–85% of all liver cancer cases globally [5]. While in Tanzania the exact incidence is unknown, a report of 142 HCC cases from Bugando Hospital in Tanzania, published in 2014 demonstrated that most HCC cases presented at a late stage and no patients received curative therapy [6]. This combination of high disease burden, the need for improved clinical services and the lack of primary data from Tanzania, motivated the TLCG to organise the first TLCC centred around the following themes:

1. To build awareness among local healthcare providers on the status of liver cancer in Tanzania and the available diagnostic and management options.
2. To update Tanzanian healthcare providers on the current standard of care for liver cancer provided in developed countries and recent advancements in liver cancer care.
3. To promote an inclusive and multidisciplinary approach in research and the clinical care of patients with liver cancer in Tanzania.

## Pre-conference activities

Prior to the conference, TLCC organisers took part in a public health campaign to raise community awareness of hepatitis B virus (HBV), the leading cause of HCC in Tanzania [6]. This included multiple media appearances with local experts discussing the risk of HBV infection and the importance of HBV vaccination and screening. The media tour culminated in a 2-day health camp, where HBV screening was provided free of charge to all community members who attended the outreach program. In total, 684 individuals were screened and 30 (4.4%) were found to be HBV positive. All screened individuals participated in one-on-one consultations with physicians to discuss HBV and learn about prevention strategies. Attendees were referred to Muhimbili National Hospital’s (MNH) Hepatitis Clinic if found to be HBV positive, and for HBV vaccination if found to be HBV negative.

## Status of liver cancer in Tanzania: epidemiology, diagnosis and treatment

After opening remarks from the Chairman of the TLCG, Dr Ally Mwanga [Muhimbili University of Health and Allied Sciences (MUHAS), Tanzania], who outlined the group’s efforts to combat liver cancer in Tanzania, the conference commenced with motivational words from the guest of honour, Dar es Salaam’s Regional Medical Officer, Dr Rashid Mfaume. Thereafter, Dr Seraphine Mrosso (MUHAS, Tanzania) and Dr John Rwegasha [Muhimbili National Hospital (MNH), Tanzania] presented on the epidemiology of liver cancer in SSA and Tanzania. They highlighted that, due to the comparative lack of liver cancer research conducted in Tanzania, figures are generally underreported and there are many unanswered questions regarding the epidemiology of the disease in the country. However, the presentation of HCC differs from that reported in Western literature with a predominance of HBV-related HCC and a considerably younger diseased population.

Following an overview of the epidemiology, there was a review of the status of screening by Dr Amani Kapinga (MUHAS, Tanzania), describing different strategies utilised in HCC screening globally and the experiences of Tanzania’s national hospital, MNH, where alpha-fetoprotein is utilised as a cost-effective solution for screening high-risk groups. This was followed by discussions on the current status of interventional radiology, surgery and medical oncology in Tanzania by Dr Erick Mbuguje (MNH, Tanzania), Dr Andrew Swallow (MNH, Tanzania) and Dr Caroline Swai [Ocean Road Cancer Institute (ORCI), Tanzania], respectively. Dr Mbuguje’s session highlighted the establishment of interventional radiology in Tanzania in 2018 in collaboration with the organisation Road2IR, as well as the specialty’s early experiences with biopsying indeterminate liver lesions and treating unresectable HCC with bland and chemoembolisation. Dr Swallow discussed the recent development of a hepatobiliary surgery program at MNH and the growth in surgical resections of both benign and malignant hepatic lesions. Dr Swai provided an overview of medical management options for advanced HCC available in Tanzania, including the national standard medication, sorafenib and the newly introduced medication, lenvatinib. Thereafter, Prof Amos Mwakigonja (MUHAS, Tanzania) provided an overview of the pathology of HCC, the histopathological appearances of different aetiologies of liver lesions, and the techniques used to differentiate these pathologies in local settings. Dr Jerry Ndumbalo (ORCI, Tanzania) concluded this part of the conference by speaking on adapting international guidelines for liver cancer to fit the resource-limited context of Tanzania. He presented his research on the availability of different treatment options across the continent and how that has aided in the development of the National Comprehensive Cancer Network Harmonized Guidelines™ for SSA.

## Liver cancer-themed scientific abstract presentations

The conference then featured liver cancer-themed abstract presentations, which included abstracts on original research and case reports. Dr Daniel Kitua (MUHAS, Tanzania) presented a comprehensive overview of in-patients treated for HCC at Tanzania’s national referral hospital. The presented findings revealed a high proportion of late-stage diseases among the study participants with only 5.6% of the participants undergoing surgery for curative intent. Dr Nazima Dharsee (ORCI, Tanzania) described the clinical presentation of patients with HCC and factors influencing survival in two tertiary care facilities in Tanzania. The presented study demonstrated a low 1-year survival rate (5%) with a median survival of 2 months. Furthermore, findings revealed that HCC-specific treatment had a positive influence on survival. Dr Latifa Abdallah (ORCI, Tanzania) presented a study on the experience of intra-arterial therapy for HCC patients in Tanzania. Findings demonstrated a 6-month post-procedural survival rate of 75% among patients receiving intra-arterial therapies. A case of HCC diagnosed in a 15-year-old male Tanzanian was reported by Dr Mwivano Shemwetta (MUHAS, Tanzania), where she highlighted the challenges of managing HCC in the paediatric population. Two rare cases of hemangiosarcoma and myopericytoma of the liver that were operated on at MNH were also presented by Dr Godbless Massawe (MUHAS, Tanzania) and Dr Novath Ngowi (MUHAS, Tanzania), respectively.

## International standards of care and the next frontier in liver cancer care

International experts were also invited to present at the meeting. Dr Victoria Chernyak (Memorial Sloan Kettering, USA) discussed the role of imaging as the primary method of diagnosis of HCC. Dr Chernyak presented a multitude of studies supporting the use of the liver reporting and data system (LI-RADS) as an effective imaging tool to risk-stratify liver lesions and diagnose HCC. She also highlighted the sensitivity and specificity of LI-RADS across different reporters and experience levels. Dr Xhorlina Marko (University of Michigan School of Medicine, USA) addressed best practices in the field of interventional radiology for the management of HCC in the US. Dr Marko provided perspectives from history on the development of different interventional management options, in addition to a glimpse into novel procedures currently in development. She included a walkthrough of different landmark trials providing evidence to the use of different procedures in current practice, such as the PREMIERE Trial [7]. Dr Marko also discussed potential barriers that might prevent the introduction of certain procedures to a resource-limited setting like Tanzania, such as various logistical challenges and substantial costs associated with radioembolisation. Dr Ashwani Sharma (University of Rochester School of Medicine, USA) presented trends in medical management of HCC with evidence from the many pivotal trials in the field, including the SHARP and IMB rave150 trials [8, 9]. Dr Ashwani discussed the rise of Atezolizumab-Bevacizumab as the standard treatment of choice at most centres in the US for unresectable HCC and about many promising medications in the pipeline.

Dr Shareef Syed (University of California San Francisco School of Medicine, USA), Dr Sonal Asthana (Aster Hospitals, India) and Dr Hossam Soliman (Menoufia University, Egypt) all provided perspectives on liver transplant surgery in their countries of practice, the US, India and Egypt, respectively. Dr Syed discussed the criteria for liver transplantation in the treatment of HCC. In addition, he also discussed the importance of a multidisciplinary treatment approach, methods of downstaging and bridging to transplant, which includes resection and local regional therapies, and other important key takeaways on the management of patients in both the pre- and post-transplant phases. Dr Asthana and Dr Soliman focused on their experiences building liver transplant programs in India and Egypt and how those lessons can be applied in Tanzania, including the importance of having a large multidisciplinary team to facilitate successful transplantation and management of associated complications. Dr Jean-Nicolas Vauthey (MD Anderson Cancer Center, USA) presented on the role of surgical resection for HCC in the US. He discussed that the vast majority of liver cancers are unresectable per international guidelines, such as the Barcelona Clinic Liver Cancer Strategy, with several factors including tumour size, severe liver fibrosis and vascular invasion contributing significantly to inoperability. However, he also highlighted that there are various interventional and medical therapies supported by research that can facilitate resection of larger and multiple lesions that would be otherwise unresectable, which is especially applicable to the Tanzanian setting, where presentation of HCC in more advanced stages is the norm. Dr Cameron Gaskill (University of California Davis School of Medicine, USA) discussed best practices to promote effective international collaboration that centres around mutual respect and understanding. Dr Gaskill reviewed the roles of different parties in facilitating international partnerships, including the benefits of tri-directional collaborations.

## Establishing multidisciplinary tumour boards and research programs in East Africa

Dr Chite Asirwa (International Cancer Institute Kenya), executive director of the International Cancer Institute (ICI), presented on ICI’s experience in supporting multidisciplinary tumour boards throughout East Africa. Dr Asirwa introduced ICI, which is based in Eldoret, Kenya, and is a leading cancer training, research and care organisation with programs across SSA. Dr Asirwa’s presentation highlighted ICI’s e-learning platform which has 2,330 users from all over the globe that participate in 69 different courses. In addition, Dr Asirwa discussed ICI’s virtual tumour boards which utilise telepathology and video conferences to allow global participation in the discussions of cancer cases from East Africa. He shared his experience on the importance of hiring a tumour board coordinator to ensure that cases are adequately shared with all relevant clinicians and closed by highlighting on the 2,096 healthcare workers that have been trained by ICI in various courses related to strengthening oncology capacity and care in East Africa as of 2020. This was followed by a mock multidisciplinary liver tumour board session organised by Dr Vijay Ramalingam (Beth Israel Deaconess Medical Center, USA) with invited guests from surgery, interventional radiology and medical oncology. This session walked through the format in which tumour boards are conducted in the US with directed questions given by the moderator designed to elicit teaching pearls on different multidisciplinary management strategies in liver cancer. Dr Mario Strazzobosco (Yale University School of Medicine, USA) provided insight from his experiences leading the Yale Liver Center, a world-renowned research centre and leader in innovation in the field. Dr Strazzabosco discussed how the framework of Yale Liver Center, structured around four core facilities (clinical and translational; morphology; cellular and molecular physiology; administrative), has facilitated the centre to publish over 400 manuscripts in high-impact journals. Dr Strazzabosco then spoke about how the centre’s structure and lessons learned from its development can be applied to the Tanzania setting to help guide future research efforts.

## Closing remarks and the way forward

Prof Andrea B. Pembe, the vice chancellor of Muhimbili University of Health and Allied Sciences, closed the conference with words of advice for the attendees on how to move forward and unite as a community in the fight against liver cancer in Tanzania. Prof Pembe commended the attendees for participating in the conference, taking an important step in improving their knowledge base in the field, and working towards a promising future where liver cancer patients in Tanzania will receive optimum care. He noted that more work needs to be done, especially in preventing patients from ever being afflicted by HCC through improved screening and vaccination programs.

## Conference impact on attendees

This inaugural conference received very positive feedback from attendees with 100% of survey respondents reporting being either ‘very satisfied’ or ‘satisfied’ with the event and 92.9% and 7.1% indicating they would ‘absolutely’ or ‘probably’ attend next year’s conference, respectively. A 12-question cross-disciplinary pre- and post-conference assessment was also completed by over 75% of attendees to assess their knowledge in the diagnosis and management of liver cancer. Median scores improved from 50% to 75% (*p* < 0.001), demonstrating the educational value of the conference among attendees.

## Conclusion

The first TLCC was designed to provide comprehensive education to the Tanzanian medical community on liver cancer and serve as a call to action for future innovation in the realms of research and care for this high-burden, high-mortality disease. This conference took a unique approach aimed at: 1) improving awareness of the current status of liver cancer care in Tanzania, 2) providing perspectives from international speakers, 3) elucidating advancements in the field and 4) proposing local practical solutions that will improve care for HCC patients and those at risk. As Tanzania’s first conference on the subject, the TLCC2023 marked an important milestone in the fight against liver cancer. A holistic approach integrating both private and public sectors is vital in improving care for patients with liver cancer in Tanzania.
